# Benchmarking Density Functional Theory for Accurate Calculation of Nitride Band Gaps

**DOI:** 10.1021/acs.jctc.5c01703

**Published:** 2026-01-27

**Authors:** Chris E. Mohn, Helmer Fjellvåg, Ponniah Vajeeston, Martin Valldor, Kristin Bergum

**Affiliations:** † Department of Chemistry and Center for Materials Science and Nanotechnology, 6305 University of Oslo, Oslo 0371, Norway; ‡ Department of Solar Energy Materials and Technologies, Institute for Energy Technology, Kjeller 2007, Norway

## Abstract

We benchmark exchange-correlation functionals for the calculation of fundamental band gaps of inorganic nitrides. These include conventional functionals such as the local density approximation (LDA), the generalized-gradient (Perdew–Burke–Ernzerhof) approximation (PBE), simple Slater exchange functionals (SLOC), specialized LDA/GGA-derived high local exchange (HLE16) and Armiento–Kümmel semilocal (AK13) functionals, meta-GGA functionals including TASK, the modified Becke–Johnson functional (mBJ), and Heyd–Scuseria–Ernzerhof (HSE06) hybrid functional, as well as quasiparticle *GW* theory. Since inorganic nitrides remain strongly under-represented in previous extensive benchmark studies, the current subdatabase contributes towards building a future large-scale balanced materials compilation of band gaps to benchmark theory. From a literature survey, we carefully collect 25 binary and 11 ternary nitrides with a focus on semiconductors spanning the periodic table, including ionic Li_3_N, antibixbyite-structured X_3_N_2_ (X = Be, Mg, Ca), early transition metals and lanthanides (e.g., ScN, YN, and LaN), ultrahard Th_3_P_4_-type structured M_3_N_4_ (M = Zr, Hf) compounds, promising photocatalysts Ta_3_N_5_, different polymorphs of III–V reference covalent nitrides (BN, AlN, GaN), and many M_3_N_4_ polymorphs (M = C, Si, and Ge) such as spinel-structured phases. Consistent with previous extensive benchmark tests, conventional LDA/PBE unsystematically largely underestimate band gaps with mean absolute errors (MAE) of >1.0 eV and mean absolute percentage errors (MAPE) of about 50%. Simple Slater exchange functional, SLOC, the GGA-derived AK13LDA and HLE16 functionals show improvement over LDA/PBE with MAE of 0.5–0.6 eV (MAPE ∼ 20–25%) with mBJ and HSE06 being the most accurate, with MAE = 0.30 and 0.28 eV (MAPE 12.1% and 11.1%), respectively. Strategies for the development of machine learning and the choice of appropriate exchange-correlation functionals for high-throughput large-scale material screening are discussed in light of these results.

## Graphical abstract

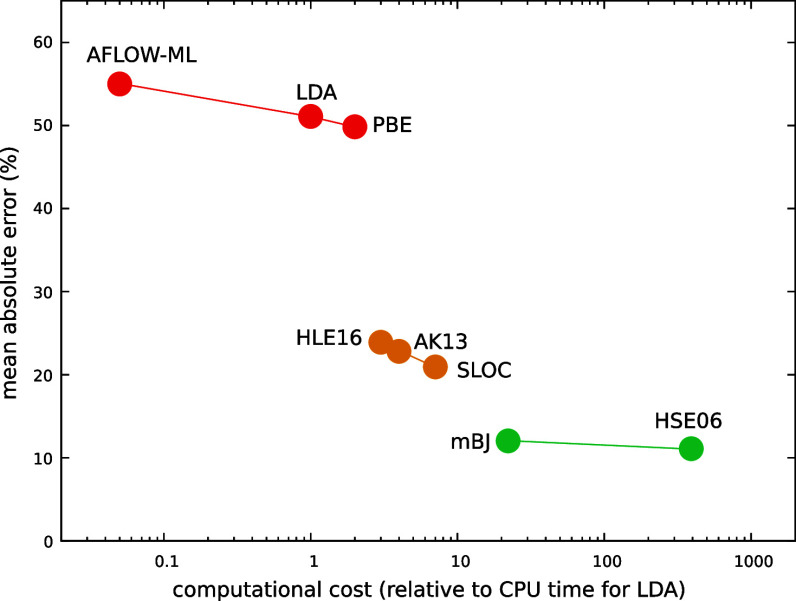

## 1 Introduction

Accurate *ab initio* calculations of material optical properties, including the fundamental band gap, *E*_g_, are essential to screen and predict new candidate materials for future solar cell technology. However, conventional implementations of Kohn–Sham density functional theory (KS-DFT)^1^—such as the local density approximation (LDA) and the generalized gradient approximation (GGA) to DFT—severely underestimate gaps. Band gaps calculated from these popular exchange-correlation functionals not only deviate from accurate experimental values by more than 1 eV (50%)^2,3^ but the errors in the calculated gaps also appear to be far from uniformly distributed throughout the periodic table, making such approximations of limited practical use for critical comparison with experiments and large-scale material screening. Even in the exact formulation of DFT, the fundamental band gap, defined as the difference between the first ionization potential and the first electron affinity, is not the same as the difference between the lowest unoccupied and highest occupied one-electron energies $\left(E_{g}^{\mathrm{KS}}\right)$ in KS-DFT. The difference, Δ_xc_, between $E_{g}^{\mathrm{KS}}$ and *E*_g_ measures the discontinuity in the exchange-correlation potential, but this discontinuity vanishes in the LDA and GGA approximations to conventional KS-DFT explaining why the LDA and GGA predictions of band gaps are far too low.^2,3^

The urge to find new functional materials has motivated researchers to develop low-cost “specialized” exchange-correlation functionals designed to accurately calculate specific properties, such as the fundamental band gap. This has led to a rich flora of available functionals developed at different levels of theory with different approximations and constraints. The library of exchange-correlation and kinetic energy functionals for DFT, LIBXC,^4^ now counts more than 500 different functionals and is rapidly growing, making it increasingly difficult to choose an appropriate functional without extensive benchmarking. Recent large-scale testing^5^ has shown that some of these exchange-correlation functionals provide a good balance between cost and accuracy in calculating band gaps. For example, band gaps calculated using the meta-GGA TASK functional and the increasingly popular mBJ functional are in much better agreement with experimental results (with an MAE of about 0.5 eV) compared to conventional LDA/GGA^6^ functionals, while being computationally much cheaper than highly accurate hybrid functionals and quasiparticle, *GW*, theory. However, since many of these promising low-cost functionals are highly parameterized and often violate important quantum mechanical and physical constraints, they need to undergo large-scale benchmarking using a chemically balanced database. Compilations used in previous extensive benchmarks of electronic structure theory, however, show that inorganic nitrides remain strongly under-represented compared to oxides, sulfides, and halides. The lack of experimental data can be explained, in part, by the difficulty in synthesizing nitrides due to the strong triple bond in elemental N_2_ that must be broken. Moreover, previous compilations used to benchmark theory have not been updated with more recent experimental band gaps for stable and metastable nitrides. Indeed, building balanced large-scale benchmarks that better incorporate nitrides is urgently needed, particularly when bearing in mind that nitrides are emerging as a highly promising class of narrow band gap semiconductors with attractive electronic and optical properties spanning a range of fields, including photovoltaics, energy storage, sensing, and detection. Theoretical large-scale predictions^7^ point to a rich fauna of metastable but kinetically highly stable phases that provide rich opportunities for the synthesis and implementation of new nitrides to supplement and replace materials in current technology. Setting up a balanced data compilation of nitrides is not only crucial to benchmark theory but is also important for further developing multiparameter functionals, such as the promising HLEXX family of exchange functionals.^8^ Moreover, the choice of appropriate functionals for efficient large-scale material screening in the search for new functional nitrides requires a cost-accuracy budget to allow for efficient large-scale material screening.

Here, we focus on semiconducting nitrides and set up a dataset of compounds with accurate band gaps reported experimentally to benchmark a range of exchange-correlation functionals to DFT and beyond. The systems selected are (nonmagnetic) semiconductors where accurate band gaps are available, but we also include a few semimetals and metals. The selection spans the periodic table and incorporates compounds with very different electronic structures. We include semiconducting Li_3_N, highly reactive metallic Na_3_N, antibixbyite-structured X_3_N_2_ (X = Be, Mg, Ca), early transition metals and lanthanides with the rock-salt structure (e.g., ScN, YN, and LaN), recently discovered ultrahard metastable Th_3_P_4_-type structured M_3_N_4_ (M = Zr, Hf) compounds, reference metal nitrides of transition metals (VN), promising photocatalysts Ta_3_N_5_, unique anti-ReO_3_ type structured Cu_3_N, different polymorphs of III–V reference nitrides (BN, AlN, GaN), and many M_3_N_4_ polymorphs (M = C, Si, and Ge) including novel high-pressure spinel phases. Ternary compounds of these binary compounds are also included in the benchmark if accurate band gaps are available. This data set of nitrides thus captures a range of different chemical environments suitable for benchmarking theory.

Using this dataset, we investigate the performance of 8 different exchange-correlation functionals including conventional (LDA, PBE) functionals, GGA-derived functionals designed to reproduce band gaps—such as the high-local exchange functional HLE16 ^8^—the simple Slater exchange potential,^9^ designed meta-GGA semilocal functionals including mBJ^10^ and TASK,^11^ traditional hybrid functionals such as HSE06,^12^ as well as quasiparticle *GW* theory^13,14^ for selected compounds. The choice of these functionals is, in part, motivated by their performance in previous extensive benchmarks where more than 20 functionals were tested.^5,15^ Therefore, we do not include other popular conventional GGA functionals, such as AMO5 and PBEsol, as they are expected to show similar performance to PBE, as demonstrated in previous extensive benchmark tests.^5,15^ Other candidate meta-GGA functionals, as well as hybrid functionals tested in refs 5 and 15 such as PBE0, B3LYP, etc., generally perform worse than mBJ/HSE06 and have therefore also been excluded from our benchmark. There are, however, several other promising families of functionals for accurate band gap calculations worth mentioning, such as the semilocal Gritsenko, van Leeuwen, van Lenthe, Baerends Solid and Correlation (GLLB-SC) functionals,^16,17^ Koopman-type functionals,^18^ recent meta-GGA functionals such as those developed by Lebeda, Aschebrock, and Kümmel,^19^ and the dielectric-dependent hybrid functionals,^20,21^ which often yield band gaps comparable in accuracy to self-consistent quasiparticle *GW* calculations but at much lower cost.

## 2 Theory and Computational Details

### 2.1 Exchange-Correlation Functionals Included in the Benchmark

The most widely used implementation of DFT is the local density approximation, which incorporates locally the exchange-correlation energy density of a homogeneous electron gas. LDA^1^ remains one of the most popular methods for calculating material properties, having successfully predicted and explained a wide range of ground-state properties of bulk materials, including their interfaces, but LDA fails to accurately calculate band gaps, as explained above. A recent implementation of LDA, SLOC,^9^ uses a simple form of the exchange potential fitted to a nonlocal Slater potential for closed-shell atoms. In spite of violating a number of conditions, including the uniform coordinate-scaling of exchange, SLOC has shown to be surprisingly promising in calculating band gaps at low cost with an MAE of 0.66 eV for a data set containing nearly 500 nonmetals.^5^ This error is less than half that of LDA and PBE.

Whereas conventional GGA (frequently implemented as the PBE functional^22^) improves ground-state energies and electron densities over LDA, both approximations yield very similar band gaps and, hence, fundamental gaps. Some recent developments of GGA designed to target band gaps include the HLE16 functional, which is derived from the Hamprecht–Cohen–Tozer–Handy (HCTH) family of functionals by means of rebalancing the weights of the exchange and correlation components of the original HCTH/407 functional.^23^ Although HLE16 neglects the constraints for the homogeneous electron gas and does not recover the limit of slowly varying densities either, it does capture the exchange scaling condition. The calculated band gaps are of comparable accuracy to those calculated using far more expensive methods with an MAE of 0.6 eV for a nearly 500-compound benchmark^5^ making it superior to PBE.

The Armiento–Kümmel semilocal energy functional (AK13)^24^ is an exchange-only approximation designed to mimic the asymptotic properties of the meta-GGA Becke-Johnson potential.^24^ For direct comparison with previous benchmarks,^5^ we add the LDA Perdew–Wang PW92 correlation term^25^ to the AK13 exchange energy. This exchange-correlation functional is thus termed AK13LDA. It is worth noting that the AK13 exchange functionals are hampered by some artificial features due to their asymptotic form, making them unsuitable for band gap calculations of semifinite systems in a similar way as the BJ and mBJ functionals (discussed below). However, for bulk materials, the asymptotic behavior of AK13 and BJ potentials is not an issue. Previous large-scale benchmarks^5^ show that AK13LDA has an MAE similar to HLE16 of ∼0.6 eV, but the MAPE is slightly higher than that of HLE16 (about 37%).

Turning to the meta-GGA classes of functionals, we also include mBJ^10^ in our benchmark. The mBJ potential is derived from the original BJ potential^26^ and is an exchange-only potential approximation, constructed to resemble the exact-exchange term from an augmented Slater potential.^27^ The mBJ exchange functional has shown remarkable success in the accurate prediction of band gaps of periodic systems, often with an accuracy similar to that of the hybrid HSE06 and quasiparticle *GW* methods. Indeed, a benchmark carried out on nearly 500 nonmetals^5^ showed that mBJ performed even slightly better than the HSE06 functional^5^ with an MAE of 0.5 eV and an MAPE of 29.6% (compared to an MAE of 0.5 eV and an MAPE of 30.8% for HSE06). The functional, however, is not a derivative of any density functional and, therefore, it violates some important exact constraints, which make it unsuitable to calculate total energies and, hence, energy derivatives such as forces for structural optimization. Moreover, since the band gap represents an average value over the entire periodic unit cell, mBJ cannot be used *as is* for systems with a vacuum gap, which currently limits its use to bulk periodic systems.

The TASK functional has been well tested for the calculation of band gaps^5,11^ and is included in our benchmark. This functional was built with the goal of introducing *ultranonlocality* similar to that of hybrid functionals but at a lower (meta-GGA level) cost. This is achieved by imposing constraints that contribute significantly to the derivative discontinuity. TASK provides a considerable improvement in the band gaps of typical semiconductors compared to other meta-GGA functionals such as SCAN^5,11^ and previous large-scale benchmark tests^5^ show that TASK performs similarly to the two GGA-derived potentials AK13LDA and HLE15 with an MAE (MAPE) of about 0.53 eV (∼37%).

Of the hybrid functionals, we include HSE06 ^12^ with 25% exact HF exchange divided into short-range and long-range terms. HSE06 has been shown to perform markedly better than other hybrid functionals such as PBE0 and HSE14 for the calculation of band gaps^15^ and has been incorporated into this benchmark to represent the performance of the more expensive hybrid class of methods.

Band gaps are also calculated for selected compounds at the quasiparticle *GW* level of theory, where *G* is the single-particle Green’s function and *W* is the screened Coulomb interaction.^13,14^ In the *G*_0_ *W*_0_ approximation, the random phase approximation is used, and the eigenvectors and eigenvalues are used directly to construct *G*_0_ and *W*_0_. “0” thus refers to “single-shot” calculations, which are nonself consistent. However, even single-shot calculations give surprisingly accurate band gaps—typically within 0.2 eV compared to accurate experiments for semiconductors and insulators^28^—but this good agreement is largely due to the fortuitous cancellation of errors due to non-self-consistency and the lack of vertex corrections. Moreover, single-shot *G*_0_ *W*_0_ may be sensitive to the starting positions (the one-electron eigensystems), and if PBE is not an appropriate starting point for quasiparticle band-structure calculations, the inclusion of exact Fock exchange might improve the quasiparticle energies. Thus, the notation *GW*@starting point (e.g., *G* *W*@PBE) is commonly used. Indeed, for some semiconductors, the discrepancy between *GW*@GGA—where GGA-derived functionals are used as starting points—may be as large as 1 eV compared to those where exact exchange is incorporated (e.g., *GW*@HSE).^29^ Although this can be overcome by full self-consistency (sc*GW* calculations), sc*GW* often worsens the band gap by 10%. Only when vertex correction, Γ, is included, the calculated band gaps are within the limit of chemical accuracy and can therefore be used to critically assess accurate experimental band gaps.^30^ Indeed, previous benchmarks using *scGW*Γ yield band gaps with similar—and often better—accuracy than that of experiment (∼0.1 eV).^30^ Since *GW* calculations scale as $O\left(N^{4}\right)$ (where *N* is the number of valence electrons), the accurate calculation of band gaps for materials with more than 12–20 atoms in the unit cell is far too expensive (even at the *G*_0_ *W*_0_ level). In addition, these calculations are extremely memory-demanding due to the dense *k*-point meshes sometimes required to ensure accurate band gaps. *GW* calculations are therefore performed only on a subset of the nitrides investigated in this work.

All calculations are carried out using VASP^22,31,32^ compiled with the LIBXC library.^4^ We used an energy cutoff of 500 eV for the electronic wave function and a density of 2π × 0.05 Å^–1^ for sampling the Brillouin zone. The electron configurations of the valence shells are as follows: Li = 1s^2^2p^1^, Be = 1s^2^2p^2^, B = 2s^2^2p^1^, C = 2s^2^2p^2^, N = 2s^2^2p^3^, O = 2s^2^2p^4^, Mg = 2s^2^2p^6^3s^2^, Al = 3s^2^3p^1^, Si = 3s^2^3p^2^, P = 3s^2^3p^3^ Ca = 3s^2^3p^6^4s^2^, Sc = 3s^2^3p^6^4s^2^3d^1^, Ti = 3s^2^3p^6^3d^2^4s^2^, V = 3s^2^3p^6^3d^3^4s^2^, Cu = 3s^2^3p^6^3d^10^4s^1^, Zn = 3s^2^3p^6^3d^10^4s^2^, Ga = 3d^10^4s^2^4p^1^, Ge = 3d^10^4s^2^4p^2^, Y = 4s^2^4p^6^5s^2^4d^1^, Zr = 4s^2^4p^6^5s^2^4d^2^, In = 5s^2^4d^10^5p^1^, Sn = 5s^2^4d^10^5p^2^, Hf = 5p^6^6s^2^5d^2^, Ta = 5p^6^6s^2^5d^3^. Calculations are carried out on the geometry obtained experimentally, and the reference is reported below and added in Table 1. For comparison with gaps calculated at the experimental geometry, we also carry out full geometry optimizations for selected compounds where all basic atomic positions, cell volume, and cell shape were allowed to relax.

Quasiparticle calculations are carried out using *G*_0_ *W*_0_@PBE for compounds with a well-defined GGA gap and *G*_0_ *W*_0_@HSE for compounds where GGA falsely predicts the semiconductors to be metals or semimetals. For *GW* calculations, we are not always able to use the same *k*-point density as for HSE06 and the other exchange-correlation functionals. The following Γ-centered meshes were used: 6 × 6 × 6 (BN (cub)), 4 × 4 × 4 (AlN (zb)), 7 × 7 × 3 (AlN (wu)) 7 × 7 × 3 (AlGaN_2_ (wu)), 3 × 5 × 4 (LiGeN), 4 × 4 × 4 (LiZnN), 3 × 5 × 4 (LiMgN) and 7 × 7 × 3 (Li_3_N). For all compounds, except LiZnN, the band gaps are converged to within 0.1 eV using these k-point grids. For LiZnN, the band gap increases by 0.2 eV when increasing the *k*-mesh from a Γ-centered 3 × 3 × 3 *k*-mesh to a 4 × 4 × 4 *k*-point mesh, but we were unable to calculate band gaps at denser grids due to a lack of computer memory, suggesting that the band gap of LiZnN may be slightly underestimated. For Li_3_N we also compared the band gaps calculated using *G*_0_ *W*_0_@PBE and *G*_0_ *W*_0_@HSE06 and found little sensitivity to the starting point in *G*_0_ *W*_0_. The band gap calculated using *G*_0_ *W*_0_@HSE06 was slightly higher by 0.15 eV compared to *G*_0_ *W*_0_@PBE. We also analyzed the convergence of the band gap with energy cutoff and found that all gaps appear to have converged to less than 0.15 eV using an energy cutoff of 500 eV.

In line with previous extensive benchmarks^15^ we do not include magnetic compounds since investigating magnetic order requires the construction of (many) different magnetic spin configurations using large supercells. These calculations, however, are extremely expensive at the hybrid levels of theory and therefore unfeasible for many of our systems with unit cells containing ∼100 atoms. VASPKIT^33^ was used for pre- and postprocessing VASP calculations.

### 2.2 Nitrides Included in the Data Set

We carried out a detailed survey of the available literature of nitride band gaps reported experimentally. Bearing in mind the exhaustive literature of available data, with relevant data not always easily accessible, there are probably important reference data missing from our data set. Although some compilations offer an extensive collection of band gaps, including halides, chalcogenides, etc.,^34^ the compilation of nitrides is often missing, making a literature survey challenging.

When different experimental results are available for a given compound, we typically select the most accurate, often the most recent, experimental data or a representative average value from an in-depth comparison and analysis of reported gaps. The precision of determining the values of *E*_g_ using experimental methods is typically about δ*E*_g_ ≈ 0.1 eV or even ≈0.01 eV, but potential systematic errors must be addressed. We therefore discuss in detail below the accuracy of the experiment and the choice of experimental band gap data, including crystallographic data used as input for the calculations. From this survey, we have selected 25 binary and 11 ternary compounds with accurate band gaps suitable for benchmarking DFT to nitrides. Calculations, in general, are performed with the geometry obtained experimentally, with a few exceptions where crystallographic data are not available. In these cases, we have used predicted values from PBE calculations (taken from the Materials Project database) and the sensitivity of the band gap to changes in the crystal structure is investigated by comparison with HSE06 since GGA tends to overestimate unit cell volumes compared to experiment.

**Li**_**3**_ **N** has at least two polymorphs. The stable polymorph at ambient conditions, α-Li_3_N, crystallizes in the hexagonal crystal system (space group *P6/mmm*)^35^ and transforms at around 1 GPa to a layered hexagonal structure, β-Li_3_N (*P6_3_/mmc*). Optical properties of α-Li_3_N, including the band gap, have been reported in ref 36, but we are unable to find data for the high-pressure phase. The absorption spectrum for the α-phase suggests an indirect band gap with two peaks arising at 2.067 and 2.104 eV below 100 K, consistent with that seen in photoluminescence and excitation spectroscopy studies, where the estimated band gap was 2.18 eV.^36^ For benchmarking DFT, a band gap of 2.10 eV is chosen, representing a reasonable average value. Band gap calculations of α-Li_3_N are carried out in the geometry obtained experimentally from ref 35 (ICSD-34280), which is consistent with other crystallographic refinements reported in ICSD (i.e., the deviation in cell volume between different reports is less than 1%^37^).

**Be**_**3**_ **N**_**2**_ has at least two polymorphs, where white α-Be_3_N_2_ has the antibixbyite structure (C-sesquioxide type).^38^ The band gap of the α-phase has been measured by reflectance ellipsometry for beryllium nitride films (grown on silicon substrates) to 3.8 eV^39^—which is slightly higher than that measured on a powder sample of cubic Be_3_N_2_ ^40^ (3.5 eV)—and taken as a reference in this benchmark. Cell volumes for different measurements reported in the ICSD database deviate by about 0.4%,^37^ and the crystal structure for band gap calculations is taken from refs 38 and 41.

**Mg**_**3**_ **N**_**2**_ also has the antibixbyite-type structure similar to α-Be_3_N_2_, with overall good consistency in the crystallographic data reported (i.e., the lattice parameters and cell volumes differ by ∼<1% between different crystallographic studies).^37^ Here we use the data reported in ref 42. Reckeweg *et al.* ^38^ reported a direct band gap of 2.8 eV by reflectance measurements (used as a reference here) in good agreement with reflectance measurements at high temperature (723 K), where band gaps of 3.15 eV (if interpreted as direct) or 2.85 eV (if indirect) were reported.^43^ Theory studies are also not in agreement with respect to the nature of the gap, but our HSE06 calculation suggests a direct gap at Γ.

**ScN** crystallizes in the rock salt structure, and the crystallographic data used in the calculation of band gaps in this work are those reported in ref 44, which is in excellent agreement with other independent crystallographic data measurements (see ICSD^37^). Several lower-level computational works and older experimental measurements are not consistent with each other, predicting indirect gaps in the range from 0 (semimetal) to 1.7 eV and direct gaps between 2.3 and 4.5 eV (see discussion in ref 45). More accurate experimental work, however, includes that of Gall et al.,^46^ who measured an indirect (fundamental) band gap of 1.3 ± 0.3 eV in good agreement with *G*_0_ *W*_0_ calculations (0.99 eV^47^ and 0.84 eV^47^). More recently, however, Al-Brithen et al. used a combination of optical absorption experiment and tunneling spectroscopy and found a slightly smaller value of 0.9 ± 0.1 eV,^48^ substantially narrowing the error bars compared to previous work. Some of the discrepancy between the experimental work in ref 47 and that in ref 46 was in part, due to a non-negligible Burstein–Moss shift associated with high charge carrier concentration as seen in ref 46. We therefore used a band gap of 0.9 eV as a reference in this study. More details and a summary of the experimental and computational work can be found in an excellent review by Biswas and Saha.^45^

**YN** crystallizes, similar to ScN, in the rock salt structure. We calculate band gaps in the crystal structure taken from ref 49, where crystallographic data (e.g., cell volumes) deviates by about 1% from other independent crystallographic measurements.^37^ Few measurements of the optical properties of YN have been reported, but Zoita et al. measured an indirect gap of 0.5 eV,^49^ which is markedly lower than those calculated from accurate hybrid calculations,^50^ as well as older optical absorption measurements, where optical band gaps of 1.5 eV^51^ and 1.45 eV^52^ were reported and used as references here. More experimental studies of band gaps would be desirable to confirm previous measurements. However, we have also included YN in our data set to discuss the origin of possible discrepancies between experiment and theory.

**LaN** also has a rock-salt crystal structure, and tens of crystallographic studies have been collected in the ICSD database, from which we have chosen that of ref 53. Hulliger^54^ measured a band gap of 0.86 eV, in good agreement with the value reported by Busch et al.^52^ (0.82 eV)—which is used as a reference in this study—and with higher-level *ab initio* theory.^50^

**TiN** has a cubic rock-salt structure^55^ possessing extensive nonstoichiometry with *x* ranging from 0.6 to 1.2 in TiN_*x*_. Stoichiometric TiN is metallic, but nonstoichiometric Ti_2_N and cubic Ti_3_N_4_ with the Th_3_P_4_-type structure, for example, are presumed to be semiconductors. For cubic Ti_3_N_4_ the band gap is reported to lie in the range of 0.8–0.9 eV,^56^ but no experimental measurements have been conducted to confirm this and therefore we have only included metallic TiN in our data set.

**Zr**_**3**_ **N**_**4**_ also belongs to a class of recently discovered metastable M_3_N_4_ phases (M = Ti, Zr, and Hf) which includes orthorhombic Zr_3_N_4_,^57^ a cubic Th_3_P_4_-type structure,^58^ and a high-pressure spinel-structured phase.^59^ In contrast to metallic ZrN, the nitrides with Zr in a “+4” valence state are all semiconductors. Optical measurements show that orthorhombic Zr_3_N_4_ has a band gap of about 1.8 eV, obtained by Tauc fitting assuming a direct band gap.^60^ This value is close to that of oxygen-bearing cubic Zr_3_N_4_ (*E*_g_ = 1.6 eV)^61^ and calculations carried out at hybrid levels of theory.^60^ We used a reference value of 1.70 eV, taken as a mean of the experimental values reported in refs 61 and 60.

**Hf**_**3**_ **N**_**4**_ phases with Hf in a valence state of “+4” also includes a high-pressure high-temperature ultrahard Th_3_P_4_-type structred phase.^58^ Moreover, genetic algorithms predict that Hf_3_N_4_ can be stable at ambient pressure and low temperature in a monoclinic *C1*2/*m1*, structure,^59^ but this has not been experimentally confirmed.^61^ The cubic Th_3_P_4_-type Hf_3_N_4_, however, has a direct gap with *E*_g_ = 1.8 eV^61^ used as a reference value here.

**VN** is a typical example of a rock-salt-structured transition-metal nitride, along with TiN, ZrN, HfN, etc. Theory is not in agreement regarding the nature of the band structure—is it half-metal or metal?—and the size and nature of the optical gap measured experimentally was estimated to be ∼3.0 eV,^62^ but the Tauc fit to (α*h*ν)^2^ vs *h*ν is not drawn in the linear region but at higher energies. Refitting indicates that the optical gap is closer to 2.5 eV. We have included VN in the data set to see if the different functionals can reproduce its electronic structure. The structural data used in the calculation are taken from ref 63.

**Ta**_**3**_ **N**_**5**_ is an important *n*-type semiconductor to benchmark theory, but different crystallographic studies propose different orthorhombic (*Pnma*, *Cmcm*) or monoclinic space groups (*C*12/*m*1). However, the differences in material density between these are small (<2%), but we have nonetheless calculated the band gaps in both crystallographic models (*C*12/*m*1 (ICSD-76460) and *Pnma* (ICSD-192986)) along with those calculated in a fully optimized crystal structure at the HSE06 level of theory to analyze the sensitivity of changes in gaps to changes in crystal geometry. Ta_3_N_5_ possesses a band gap of 2.1 eV^64^ estimated from the UV–vis diffuse reflectance spectrum on a powder sample, which is used as a reference in our *ab initio* band gap calculations.

**Cu**_**3**_ **N** has attracted attention among TM-rich nitride semiconductors due to its unique defect-tolerant anti-ReO_3_ crystal structure (*Pm3̅m*)^65^ characterized by corner-sharing Cu–N octahedra embedded in a cubic lattice. Person^66^ measured an optical gap of 0.25–0.83 eV for Cu_3_N thin films grown using reactive RF magnetron sputtering, which was markedly higher than that in previous experiments (see e.g., ref 67). It was suggested that this discrepancy in gaps is due to the sensitivity of deposition conditions, providing a plausible explanation for the low and variable electrical resistivity measured. However, since relevant optical data are unavailable (ref 67), we are unable to confirm this. By contrast, the surprisingly large gap of 1.8–1.9 eV reported in ref 67 is possibly due to extensive nonstoichiometry since the band gap is sensitive to changes in Cu^+^ content. These values are also in better agreement with that of Birkett et al.^68^ who reported a slightly lower direct band gap of 1.68 eV at 300 K using spectroscopic ellipsometry on thin-film powders. Other band gaps reported in the literature are constrained by these studies and are fairly consistent with gaps in the range of 1.3–1.6 eV.^69–72^ Here we use a recent optical band gap of 1.4 eV^69^ —commonly used as a reference for Cu_3_N band gaps^70,73^ —and discuss this choice in light of our calculated band gaps.

**Zn**_**3**_ **N**_**2**_ crystallizes in the cubic Hausmannite-like structure and remains a reference to benchmark theory. Calculations of the band structure, including gaps in this work, are performed using the crystal structure taken from ref 74. Most measurements of intrinsic band gaps report band gaps in the range of 1.0–1.25 eV.^75–77^ The small discrepancy between these measurements is probably explained by crystallinity and possibly the contamination of oxygen. We used an average gap of 1.10 eV, which is in good agreement with quasiparticle *GW* calculations on bulk Zn_3_N_2_ ^78^ (*E*_g_ = 1.2 eV). The estimated band gap of ∼3.2 eV in an early study^79^ is, therefore, probably associated with the formation of zinc oxide.^75^

**BN** exists in a range of polymorphs, including hexagonal, cubic, and wurtzite-structured forms, where the hexagonal phase is thermodynamically stable. Several experimental and computational studies of optical properties, including band gaps, have been carried out on all polymorphs. Cassabois et al.^80^ accurately determined the band gap of hexagonal BN to be 5.955 eV using optical spectroscopy in excellent agreement with the luminescence excitation spectroscopy experiment of Evans et al. (5.96 ± 0.04 eV)^81^ (used as a reference here). The band gap of cubic boron nitride is also accurately determined as 6.36 ± 0.03 eV^81^ (used as a reference in this work) consistent with older optical measurements^82^ (6.1 ± 0.2 eV), ultraviolet absorption spectra (>6.4 ± 0.5 eV),^83^ and soft X-ray spectroscopy (6.0 ± 0.5 eV).^84^

**AlN** also has a wurtzite-type structure, a metastable zinc-blende phase, and a high-pressure rocksalt structured phase. Wurtzite-structured AlN has been extensively studied both experimentally and computationally and remains a reference to benchmark theory. The AlN band gaps are consistently reported to be in the range of 6.0 and 6.3 eV.^85–87^ Guo and Yoshida,^86^ for example, report a band gap of 6.026 eV at 300 K,^86^ which is in good agreement with those of Yamashita et al.^85^ (6.23 eV at 300 K) and those of ref 87 (6.2–6.3 eV), assuming an exciton binding energy of 70–80 meV. We use a mean value of these as an estimate of the fundamental band gap obtained experimentally (i.e. *E*_g_ = 6.25 eV). Yamashita et al.^85^ also showed that temperature effects on gaps are only about 0.03 eV when the temperature is increased from 77 to 300 K. Much less attention has been drawn to zinc-blende-structured AlN. Analysis of the extinction coefficient from thin-film depositions, however, shows that AlN with the zinc-blende structure is a semiconductor with an indirect band gap of ∼5.34 eV.^88^

**GaN** has, similar to BN and AlN, a wurtzite-type structure,^89^ a metastable zinc-blende β-phase,^90^ and a high-pressure cubic spinel-structured phase. GaN has been extensively studied theoretically, and several experiments report a band gap in the range of 3.3–3.5 eV for GaN wurtzite (e.g., see ref 91). While Ref 91 report a gap of 3.39 eV at 300 K, Monemar^92^ used photoluminescence excitation spectra and found a fundamental band gap of $3.503_{-0.002}^{+0.005}$ eV at 1.6 K with a small exciton binding energy of $28_{-3}^{+6}$ meV in good agreement with that of Muth et al.^93^ who measured a band gap of 3.452 eV at 293 K with an exciton binding energy of ∼20 meV. We use a band gap of 3.45 eV as a reference value of the fundamental band gap in this work. The band gap of zinc-blende, which is slightly less than that of wurtzite-structured GaN, consistently lies in the range of 3.2–3.3 eV as measured by different experimental studies,^90,91,94^ and we have used a mean value of these (3.25 eV).

**InN** has, in line with the other boron-group nitrides, a stable wurtzite-type structure with a band gap of ∼0.7 eV.^95,96^ Although estimates of the band gap from older studies were much higher (>1.5 eV),^95^ a value of around 0.7 eV is widely accepted as reasonable for intrinsic InN with a gap value of 0.6 eV providing a lower bound estimate as suggested by the shift in the PL peak position and the band gap shrinkage associated with conduction-band renormalization.^97^ Zinc-blende structure InN has a very similar band gap of 0.6 eV^98^ estimated from the PL of an MBE-grown film.

**C**_**3**_ **N**_**4**_ has a rich polymorphism^99^ and at least seven different phases exist, including cubic C_3_N_4_, “pseudo-cubic” C_3_N_4_, α-C_3_N_4_, β-C_3_N_4_, and various forms of graphitic (g-C_3_N_4_) phases, such as graphitic-*h*-heptazine, graphitic-*o*-triazine, and graphitic-*h*-triazine. Of these, graphitic C_3_N_4_ (with tri-*s*-triazine C_6_N_6_ building blocks) has been the most extensively studied phase, both computationally and experimentally. Two different experimental studies are in good agreement with the estimated gaps of 2.75 eV^100^ and 2.70 eV^101^, and a mean value of these two is used as a reference.

**Si**_**3**_ **N**_**4**_ has at least three crystalline polymorphs, including a trigonal α-Si_3_N_4_ (space group *P*31*c*) phase, a hexagonal β-Si_3_N_4_ (space group *P*6_3_) phase, and a high-pressure cubic spinel-structured γ-phase. There are few accurate determinations of band gaps of the ambient pressure polymorphs, but optical properties for the spinel phase have been accurately determined by Museur et al.^102^ who measured a gap of 5.05 eV ± 0.05 eV (used as a reference value in this work) with a surprisingly high exciton binding energy of 0.65 eV. This gap is in good agreement with that reported by Boyko et al.,^103^ which is 4.8 ± 0.2 eV—albeit with a markedly smaller exciton binding energy of 0.33 eV—and also consistent with gaps from mBJ and *G*_0_ *W*_0_ calculations reported in ref 103 and ref 104, respectively. Crystallographic data from the high-pressure spinel phase is taken from an XRD study.^105,106^ We carefully investigated geometric effects on band gaps since a variation in the lattice parameter of 3% has been shown to shift the band gap energy by 0.64 eV.^105^

**Ge**_**3**_ **N**_**4**_ also has, in addition to the ambient pressure α- and β-phases, a high-pressure cubic spinel-structured γ-phase that is formed above >12 GPa and >400 K.^107^ Although there are a few experimentally reported band gaps for the α- and β-phases, the accuracy of these is uncertain, and we focus here on the γ-phase, where Feldbach et al. have recently accurately determined the band gap to be 3.65 ± 0.05 eV.^108^ We use this value as a reference for band gap investigations, and crystallographic data are taken from ref 107.

**LiMgN** is orthorhombic (space group *Pnma*) at room temperature but undergoes a phase transition above ∼670 K to a simple cubic antifluorite-structured phase (space group *Fm3̅m*). The possibility of a disordered cation sublattice has been suggested^109^ since XRD measurements were unable to fully resolve the cation positions.^109^ The band gap of the low-temperature phase of LiMgN was measured by photoacoustic spectroscopy and found to be 3.2 eV, supported by an optical transmission spectrum.^109^

**LiZnN** is cubic (space group *F* *4̅3m*)^110^ with a band gap of 1.98 eV^111^ measured using photoacoustic spectroscopy on a deep red polycrystalline sample. This value is in good agreement with the optical band gap of 1.91 eV reported from a Tauc plot assuming a direct transition.^112^ In this work, we use a mean value of the band gaps reported in these two experimental studies (1.95 eV) as a reference.

**LiGe**_**2**_ **N**_**3**_ crystallizes in a superstructure of wurtzite (space group *Cmc*2_1_) where the cations are fully ordered.^113^ The band gap of 4.16 eV was estimated from a Tauc plot assuming a direct transition.^113^

**MgSiN**_**2**_ and **MgGeN**_**2**_ both have wurtzite-like structures (space group *Pna*2_1_)^114,115^ and optical band gaps of 4.8 and 3.2 eV, estimated from diffuse reflectance spectra using the Kubelka–Munk function.^116^

**ZnSnN**_**2**_ probably has a wurtzite-like structure (space group *Pna*2_1_) as a thermodynamically stable phase at room temperature, but depending on experimental conditions, a zinc-blende phase may also form, possibly with extensive kinetic (frozen-in) disorder depending on deposition rate and temperature.^117^ The presence of two nearly degenerate crystal structure^117^ is also confirmed by theory.^117^ Here, we calculate the band gap within the *Pna*2_1_ space group. The band gap is also tunable depending on the deposition parameters—inducing various degrees of disorder. This makes a comparison between different experimental and computational studies difficult, also hampered by a substantial Burstein–Moss shift.^155^

**AlGaN**_**2**_ has the Lavarevićite-type structure and crystallizes in the trigonal *P*3̅*m*1 space group, according to *ab initio* DFT calculations (MP-1228894).^118^ We are, however, unable to find crystallographic data from the experiment, and the band gap has been estimated from the bowing parameter by interpolation from their end-members GaN and AlN^119^ using *E*_g_(*A*_1 – *x*_ *B_x_N*) = (1 – *x*)*E*_g_(*A*) + *xE*_g_(*B*) – *x*(1 – *x*)*C*, where *C* is the bowing parameter taken from ref 116.

**InAlN**_**2**_ crystallizes in the orthorhombic *Pna*2_1_ space group (MP-1247201)^118^ and similarly to InAlN_2_ the band gap (2.89 eV) has been estimated from interpolation of the AlN and InN end-members using the bowing parameter taken from ref 116.

**InGaN**_**2**_ is theoretically predicted at the GGA level to crystallize in the trigonal *P*3*m*1 space group (Enargite-like structure) with a band gap calculated using the bowing parameter taken from ref 116. We reoptimized the crystal structure at the HSE06 level to analyze the sensitivity of the band gap to changes in the geometry.

**CaSiN**_**2**_ has both a low-pressure phase^120^ and several high-pressure modifications predicted by theory,^121,122^ including a β-phase that has been synthesized.^121^ Different experimental studies also report different values of the band gaps for the low-pressure α-phase. While ref 123 reports a gap of 3.95 ± 0.30 eV from soft X-ray absorption and emission spectroscopy measurements, Groen et al.^124^ reported a markedly higher gap of 4.5 eV using diffuse reflectance spectroscopy. The reasons for the discrepancy are unclear; possible explanations may be due to the very different techniques used.^123^ Here, we use the gap reported in ref 124 as it is more consistent with theory, which we also turn to discuss below.

**CaMg**_**2**_ **N**_**2**_ has the same structure as its end-members Ca_3_N_2_ and Mg_3_N_2_ namely the cubic antibixbyite structure (space group *Ia*3̅).^41^ Optical properties have been measured on powders using IR reflectivity measurements, and the band gap is estimated to be 3.25 eV from a Tauc plot^38^ (used as a reference in the benchmark here).

There are several interesting families of nitrogen-containing compounds that have not been included in our data set such as azides. These include, for example, alkali metal azides (e.g., NaN_3_, KN_3_, RbN_3_) and explosive AgN_3_ silver azide. The reason for excluding these is not only because accurate experimental data are scarce but also because the nature of the nitrogen bonds is very different from the other inorganic nitrides investigated in this study since nitrogen forms molecular unit of $N_{3}^{-}$ (azide) ions.

We have discussed above possible discrepancies arising from the definition of band gaps since KS-DFT calculates the HOMO–LUMO gap between one-electron functions that deviates not only from the quality of the functional but also from the discontinuity, Δ_*xc*_, in the exchange potential due to the difference in the fundamental gap and the HOMO–LUMO gap. Other possible discrepancies between experiment and theory that must be addressed for a critical comparison include electron–phonon interaction (EPI), such as zero-point phonon renormalization (ZPR),^125^ exciton binding energy (i.e., electron–hole interaction), and band tails associated with configurational disorder and defects. EPI is, generally, less than 0.1 eV—particularly when compared with experiments carried out at room temperature—and can be calculated, for example, using density-functional perturbation theory (DFPT).^126^ Calculation of exciton binding energies for the direct comparison with the optical band gap can be estimated by setting up the Bethe–Salpeter Hamiltonian from single-particle quasi-energies calculated at the GW level. Contributions to exciton binding energy are in most of our cases less than 0.1 eV, but for wide-gap semiconductors they may be non-negligible for a critical evaluation of experiments. For Th_3_P_4_-structured cubic nitrides investigated in this work, for example, the exciton binding energy is between 0.032 and 0.046 eV, but for Si_3_N_4_ the exciton binding energy was found to be as large as 0.3–0.6 eV.^102,103^ Band tails with localized bands due to disorder may also lower the band gap by a few tenths of an eV, depending on the nature of disorder and can be calculated from ensemble averages using Monte Carlo techniques and thermodynamic averaging by sampling different atomic configurations or defect states.^127,156^ Phonon–electron coupling, exciton binding energy effects, and band tails are all discussed as potential sources of discrepancy between experiment and theory.

## 3 Results and Discussion

**Table 1:** Collection of Band Gaps (in eV) Calculated Using Different Exchange-Correlation Functionals , as well as Experimental Results^a^

Compound	EXP	LDA	SLOC	PBE	HLE16	AK13	mBJ	TASK	HSE06	Gap ref.	Structure ref.	Structure ID
Li_3_N	2.10	1.14	2.65	1.04	1.72	1.60	2.28	2.00	1.95	^36^	^35^	ICSD-34280
α-Be_3_N_2_	3.80	3.06	4.85	3.11	3.95	4.22	4.53	4.32	4.33	^39^	^38^	ICSD-412667
Mg_3_N_2_	2.80	1.68	2.68	1.63	2.26	2.54	2.90	2.95	2.71	^38^	^42^	ICSD 425109
α-Ca_3_N_2_	1.90	1.09	2.77	1.11	2.48	2.72	2.68	2.42	2.02	^128^	^41^	ICSD-410754
ScN	0.90	0.00	0.35	0.00	0.36	0.67	0.89	0.81	0.91	^48^	^44^	ICSD-644666
YN	1.45	0.09	0.94	0.18	0.89	0.97	1.11	1.13	1.11	^51^		ICSD-644873
LaN	0.82	0.00	0.29	0.00	0.92	n.c	0.84	n.c	0.81	^54^	^53^	ICSD-641467
TiN	0.00	0.00	0.00	0.00	0.00	0.00	0.00	0.00	0.00		^55^	ICSD-126465
Zr_3_N_4_	1.70	0.96	1.13	1.03	1.15	1.45	1.59	1.74	1.93	^60^	^57^	ICSD-78944
Hf_3_N_4_ (*I*43*d*)	1.90	0.89	1.15	1.01	1.14	1.55	1.93	1.69	1.92	^61^	^58^	ICSD-97997
VN	0.00	0.00	0.00	0.00	0.00	0.00	0.00	0.00	0.00		^63^	ICSD-644858
Ta_3_N_5_ (*c*12*m*1)	2.10	1.40	1.40	1.52	1.30	1.94	2.40	2.21	2.44	^64^		ICSD-76460
Cu_3_N	1.40	0.24	1.48	0.22	1.00	0.49	0.27	0.09	0.98	^70^	^65^	ICSD-167835
Zn_3_N_2_	1.10	0.00	1.12	0.00	0.73	0.18	0.74	0.05	0.86	^75–77^	^74^	ICSD-84918
BN (hex-g.)	5.96	4.04	5.51	4.24	5.23	5.35	6.15	6.09	5.55	^80,81^	^129^	ICSD-240996
BN (cub)	6.36	4.34	6.20	4.46	5.34	5.63	5.80	5.39	5.80	^81^		ICSD 86357
AlN (wu)	6.25	4.24	5.14	4.21	4.86	5.41	5.37	5.89	5.58	^85–87^	^89^	ICSD 34475
AlN (zb)	5.37	3.22	5.07	3.32	4.44	4.47	4.82	4.62	4.57	^88^		MP-1700
GaN (wu)	3.45	1.93	2.83	1.91	2.80	2.74	3.10	2.60	3.19	^93^	^89^	ICSD-34476
GaN (zb)	3.25	1.69	2.59	1.67	2.53	2.44	2.82	2.30	3.36	^90,94^	^90^	
InN (wu)	0.78	0.00	0.76	0.00	0.67	0.50	0.84	0.31	0.71	^95,96^	^130^	ICSD-109463
InN (zb)	0.61	0.00	0.61	0.00	0.48	0.28	0.65	0.02	0.91	^98^	^98^	
g-C_3_N_4_	2.73	1.05	1.82	1.19	1.58	1.64	2.95	2.28	2.74	^100,101^		ICSD-971684
γ-Si_3_N_4_ (hp)	5.05	3.32	4.54	3.46	4.25	4.63	4.81	4.97	4.80	^102^	^106^	
γ-Ge_3_N_4_ (hp)	3.65	2.07	2.00	2.69	2.37	2.78	3.86	4.18	3.91	^108^	^107^	ICSD-87767
LiZnN	1.95	0.73	1.93	0.67	1.58	1.17	1.82	1.33	2.39	^111,112^	^110^	ICSD-16790
LiMgN	3.20	2.32	3.13	2.25	2.75	3.17	3.55	3.53	3.92	^109^	^131^	ICSD-167574
LiGe_2_N_3_	4.16	2.91	2.82	2.93	3.22	3.77	4.08	4.15	4.88	^113^	^113^	ICSD-129689
MgSiN_2_	5.84	4.14	5.04	4.15	4.92	5.61	5.51	5.97	6.02	^116^	^115^	ICSD-30730
MgGeN_2_	3.20	2.01	2.36	2.04	2.53	2.89	3.23	3.15	3.84	^116^	^116^	ICSD-433632
ZnSnN_2_	1.40	0.29	1.38	0.29	1.25	0.93	1.27	0.78	2.00	^117^	^117^	
AlGaN_2_	4.65	2.90	3.78	2.88	3.69	3.78	3.98	3.93	4.74	^119^		MP-1228894
InGaN_2_	1.69	0.23	1.08	0.22	1.00	0.91	1.32	0.79	1.72	^119^		MP-1223660
AlInN_2_	2.89	1.51	2.26	1.51	2.15	2.27	2.52	2.44	3.13	^119^		MP-1247201
CaSiN_2_	4.50	3.14	3.44	3.28	3.86	4.27	4.43	5.05	5.00	^124^	^121^	ICSD-433179
CaMg_2_N_2_	3.25	2.08	2.86	2.06	2.68	3.21	3.42	3.66	3.13	^38^	^41^	ICSD-411175

a Runs labeled “n.c” indicate that the wave function did not converge after 400 electronic steps also when "robust" conjugent algorithms for the electronic optimizations were used For AlGaN_2_, InGaN_2_, and AlInN_2_, the band gap is calculated from those of the end members using the formula *E*_g_(*A*_1 – *x*_ *B_x_N*) = (1 – *x*)*E*_g_(*A*) + *xE*_g_(*B*) – *x*(1 – *x*)*C,* where *C* is the bowing parameter obtained experimentally.^119^

The calculated band gaps are compiled in Table 1 together with available experimental data, and plots of experimental versus calculated band gaps are shown in Figure 1. In Table 2, we collect statistics of the different functionals including absolute errors (ME, MAE), relative errors (MPE, MAPE), number of false metals, number of band gaps that deviate less than 0.5 eV from those measured experimentally, standard deviations for a linear fit, and *y* = *ax* + *b* to the data for a given functional. It is worth bearing in mind that small band gap materials are strongly weighted in the calculation of MAPE, i.e., a small discrepancy in the calculated band gap may lead to large percentage errors. MAPE/MPE should therefore be used together with parameters that measure the *absolute* errors (e.g., ME/MAE) when discussing the accuracy of theory. The CPU time taken (relative to that used for the LDA set) is also collected to measure the cost of each functional, an important parameter in a cost-accuracy budget for large-scale material screening.

**Figure 1:**
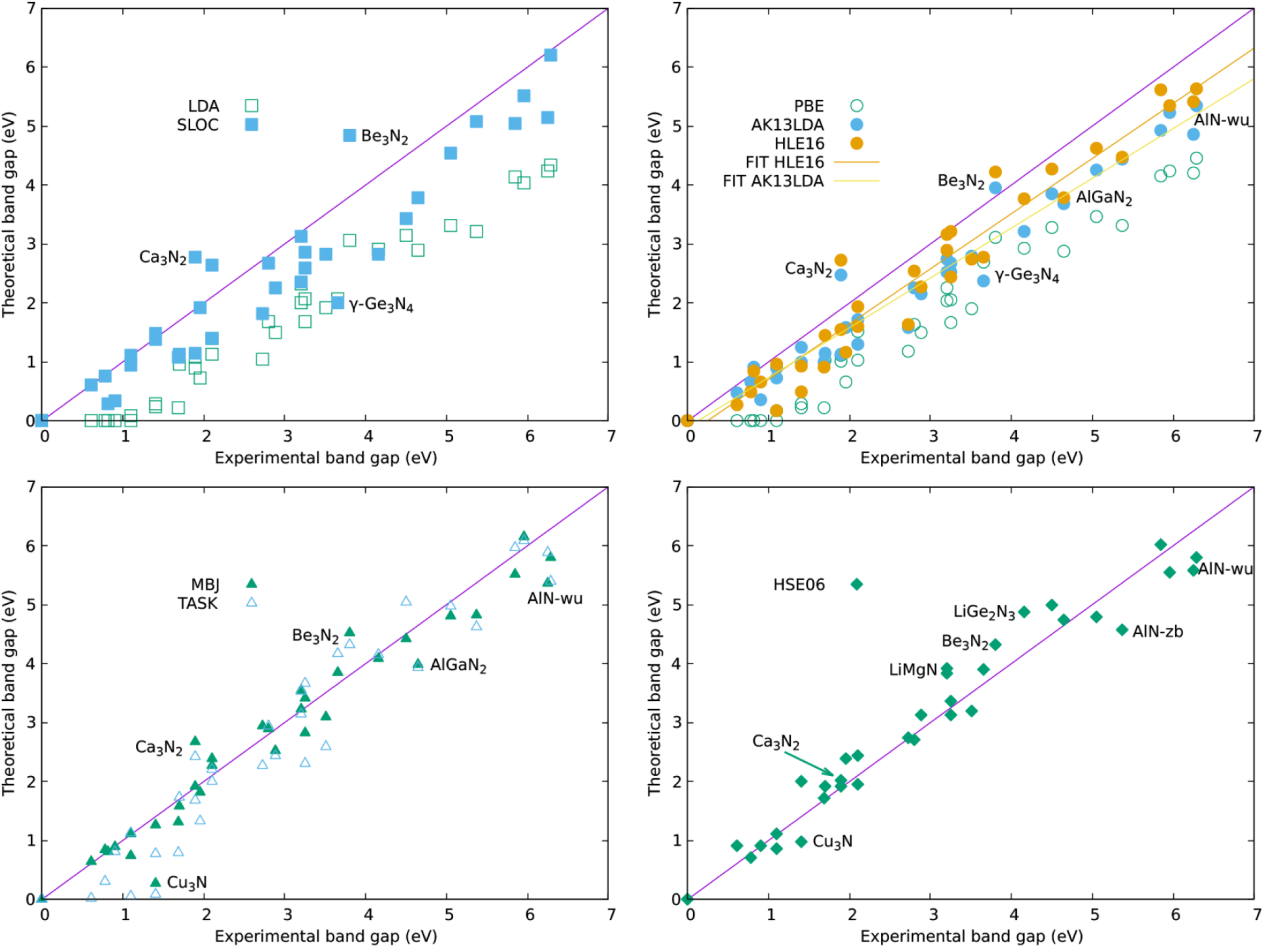
Plot of experimental vs calculated band gaps for 36 nitrides. Top left and top right figures are results from LDA-derived and GGA-derived semilocal functionals, whereas bottom left and bottom right are results from meta-GGA and hybrid functionals. Linear fits *y* = *ax* + *b* to the AK13LDA and HLE16 data are shown with (*a*_HLE16_ = −0.229, *b*_HLE16_ = 0.936 and *a*_AK13_ = −0.108, *b*_AK13_ = 0.845).

**Table 2:** Statistical Errors of the Calculated Band Gaps with Different Functionals, with the Experimental Data (36 Compounds) as a Reference^a^

Functional	ME (eV)	MAE (eV)	MPE (%)	MAPE (%)	False metals	*N* |*y_i_* – *y*_*i*, exp_| < 0.5 (eV)	σ (eV)	CPU time
LDA	1.22	1.22	51.0	51.0	5	0	0.50	1
PBE	1.17	1.17	48.4	49.8	5	0	0.45	2
HLE16	0.57	0.62	20.1	23.2	0	12	0.41	3
AK13LDA	0.44	0.51	19.2	22.9	0	20	0.40	4
SLOC	0.41	0.55	15.8	21.0	0	13	0.55	7
TASK	0.25	0.45	16.2	22.5	0	20	0.51	38
mBJ	0.11	0.30	4.6	12.1	0	30	0.39	22
HSE06	–0.04	0.28	–2.7	11.1	0	30	0.36	390

a The mean error $\mathrm{ME}=\overset{n}{\underset{i=1}{\sum }}\left(y_{i}-y_{i,\exp}\right)/n$ , the mean absolute error **MAE** = $\overset{n}{\underset{i=1}{\sum }}\left|y_{i}-y_{i,\exp}\right|/n$ , the error standard deviation STD = $(\overset{n}{\underset{i=1}{\sum }}(y_{i}-y_{i,\exp}-\mathrm{ME}{)}^{2/n}{)}^{1/2}$ , the mean percentage error MPE = $100\times \overset{n}{\underset{i=1}{\sum }}\left(y_{i}-y_{i,\exp}\right)/\left({ny}_{i,\exp}\right)$ , the mean absolute percentage error $\mathrm{MAPE}=100\times \overset{n}{\underset{i=1}{\sum }}\left|y_{i}-y_{i,\exp}\right|/(ny_{i,\exp})$ , and the standard deviation of the linear fit (*y =* *ax* + *b*) to the bandgaps for a given functional. CPU time is the overall CPU time used for the test set (relative to the total time for LDA) on an Intel E5-2683v4 2.1 GHz Linux cluster using VASP-input parameters as described in the section Theory and Computational Details.

### 3.1 Accuracy and Performance of Different Functionals

Consistent with previous benchmarks,^5,15,30,132,133^ LDA and PBE severely underestimate band gaps for nitrides, with MAE (MAPE) of 1.22 eV (51%) and 1.17 eV (50%), respectively, which is explained by the well-known vanishing discontinuity in the exchange-correlation potentials. Several of the band gaps calculated using LDA and PBE for small band gap semiconductors, such as ScN, LaN, Zn_3_N_2_, and InN, are thus falsely predicted to be metals or semimetals.

The simple SLOC potential has surprisingly small errors. With an MAE of 0.55 eV and an MAPE of 21%, it performs far better than both LDA and PBE, possessing similar accuracy as the GGA-derived HLE16 and AK13LDA potentials, which are both designed to target band gaps. In contrast to LDA and PBE, SLOC also correctly predicts all ScN, LaN, Zn_3_N_2_, and InN to be semiconductors. However, the experimental vs SLOC-calculated band gaps (top left, Figure 1) are markedly more scattered compared to the other functionals. This is also reflected in the standard deviation (σ = 0.55 eV), which is the largest of all functionals investigated, illustrating that a very simple form of the exchange functional, *an* ^*b*^ of the density *n* with only two adjustable parameters *a, b* ^9^ struggle to capture very different electronic structures. Sensitivity to changes in the band gap due to changes in *a* and *b* was investigated in ref 5 by refitting to minimize MAPE for a large number of band gaps but only a small decrease MAPE was found compared to the original data, suggesting limited room for improvement.

The two specialized GGA-derived potentials, HLE16 and AK13LDA, perform much better than PBE itself, with MAE (MAPE) of 0.62 eV (23%) and 0.51 eV (23%), respectively. The markedly better performance of these two functionals compared to that of PBE is not surprising since they were developed to target band gaps, but the main reason for their good performance is that they do not predict any of the small band gap semiconductors to be metals, thereby improving the overall statistics. However, both tend to slightly underestimate the band gaps. This is clear from the plot in Figure 1, as well as a linear fit to the HLE06 and AK13 gaps visualized in Figure 1 that deviate marginally from *y* = *x*.

Turning to the two meta-GGA-derived functionals investigated in this work (mBJ and TASK), both perform better than the LDA- and GGA-derived ones. mBJ performs markedly better than TASK, consistent with previous extensive benchmarks.^5^ Indeed, with an MAE of 0.30 eV and an MAPE of 12%, mBJ provides a highly accurate prediction of band gaps, similar to that of HSE06. However, both mBJ and TASK are hampered by the slow convergence of the wave function. Although the use of *GW*-developed potentials seems to speed up the overall convergence of the electronic wave function for mBJ and TASK, 10 times more electronic steps (on average) are needed compared to LDA- and GGA-derived functionals, which explains why these are about an order of magnitude more expensive than LDA and PBE. HSE06 performs best of all functionals, although marginally better than mBJ, with an MAE of 0.28 eV and an MAPE of 11%, but is computationally more than one order of magnitude more expensive than mBJ.

### 3.2 Assessment of Experimental Band Gaps

Several distinct anomalies are marked in Figure 1 such as Ca_3_N_2_ and Be_3_N_2_ where all SLOC, AK13LDA, HLE16, TASK, and mBJ-calculated band gaps overshoot those reported experimentally. For Ca_3_N_2_, however, HSE06 deviates by only 0.1 eV from the undocumented experimental value of 1.9 eV reported in ref 128, which could indicate that the direct band gap of 1.55 eV estimated from a Tauc plot may be too low.^41^ Due to oscillations in the region where a linear extrapolation can be drawn in the (α*h*ν)^2^ vs *h*ν Tauc plot, plausible extrapolations suggest band gaps in the range of 1.4 eV to about 1.6 eV. However, even a high-end estimate of the optical band gap is surprisingly low compared to our mBJ and HSE results, as well as the gap reported in ref 128. A possible explanation could be due to exciton binding energies, but although this rarely exceeds 0.1 eV for inorganic nitrides, there are exceptions, as illustrated above for some wide-gap semiconductors such as γ-Si_3_N_4_. However, calculation of exciton binding energies from the Bethe–Salpeter equations using single-particle quasi-energies calculated at the *GW* level is computationally unfeasible for Ca_3_N_2_ and Be_3_N_2_ because of the large unit cells containing 80 atoms. The band gap of Be_3_N_2_ calculated using HSE is also higher than that reported from a Tauc plot to the absorption spectrum, but without confirmations from higher levels of (quasiparticle) theory—which again is computationally unfeasible—it is difficult to draw firm conclusions about the accuracy of the band gap obtained experimentally in ref 38.

The band gap of Cu_3_N is also markedly underestimated at the meta-GGA and hybrid levels of theory, and in particular, the very small band gaps from TASK and mBJ are surprising because the large-scale material benchmark in ref 15 suggests that all functionals show very similar performance over *d* and *sp* subdata sets. The band gap calculated using HSE06 (0.98 eV) improves upon the experimental band gap, but our calculated *G*_0_ *W*_0_@HSE06 value of 1.78 eV (see Table 3) is markedly higher than the reference used here, but it is in good agreement with the experimental value reported in ref 67 and a more recent study.^68^ Accurately determining the band gaps of Cu_3_N remains a challenge, with a range of values suggested from optical measurements of thin films. The sensitivity of the band gap to changes in stoichiometry, deposition conditions, and film thickness, inducing various degrees of material strain, also makes a direct comparison with periodic *ab initio* theory challenging.

**Table 3:** Calculated *GW* Band Gaps from This Work and a Selection from the Literature^a^

Compound	Exp/eV	*G*_(0)_ *W*_0_@DFT/eV	sc*GW*/eV	sc*GWT*/eV
Li_3_N	2.10	1.88		
ScN	0.89	1.14*^b^* ^1^, 0.84*^b^* ^2^		
Cu_3_N	1.40	1.78, 1.00*^c^* ^1^, 0.26*^c^* ^2^		
BN (cub)	6.28	6.27, 6.66*^f^* ^1^	7.14*^f^* ^2^	6.59*^f^* ^3^
AlN (wu)	6.25	5.99, 5.72*^h^* ^1^, 6.35*^h^* ^2^,6.47^i^	6.77^j^, 6.80^k^	
AlN (zb)	5.37	4.92	5.64^*j*^	
GaN (wu)	3.45	3.30*^f^* ^1^, 3.16^*d*^, 3.24^i^	3.82*^f^* ^2^	3.27*^f^* ^3^
GaN (zb)	3.25	3.24^i^		
Li_3_N	2.10	1.88		
LiMgN	3.20	3.63		
LiZnN	1.95	1.69		
AlGaN_2_	4.65	4.39		

a Here, we use *G*_0_ *W*_0_@PBE for compounds where PBE correctly predicts them to be semiconductors and *G*_0_ *W*_0_@HSE for compounds where PBE falsely predicts semiconductors to be metals or semimetals. Previous *GW* calculations for ScN are single-shot *G*_0_ *W*_0_@LDA (*b*1) and *G*_0_ *W*_0_@OEPx­(cLDA) (*b*2), where OEPx­(cLDA) refer to exact-exchange optimized effective potential as initial single-particle eigenvalues for *G*_0_ *W*_0_ calculations.^47^ *c*1 is taken from ref 69 using the energy-only self-consistent *GW*_0_@PBE+U possibly with HSE as the starting point for quasiparticle calculations, and *c*2 is taken from ref 152 using *GW*_0_@DFT+U. *f*1, *f*2, and *f*3 are *GW*_0_@DFT, sc*GW*, and sc*GW*Γ (with vertex correction in *W* only) calculations taken from ref 30. *h*1 and *h*2 are *G*_0_ *W*_0_@PBE and *G*_0_ *W*_0_@HSE06 taken from ref 29 . *i* is *G*_0_ *W*_0_@OEPx­(cLDA) calculations taken from ref 157 whereas d ,j and *k* are taken from ref 153, ref 137, and ref 158, respectively.

Although most of the band gaps for the two most accurate methods, mBJ and HSE06, deviate by less than 0.5 eV compared to the experiment (see Table 2), there are a few larger deviations that must be addressed for a critical evaluation/assessment of experimental data and theory. The largest underestimates of HSE06 are LiMgN and LiGe_2_N_3_, followed by MgGeN_2_ , α-Be_3_N_2_, CaSiN_2_ and LiZnN where HSE are lower by 0.72 eV, 0.72 eV, 0.64 eV, 0.53 eV, 0.50, and 0.44 eV, respectively (see Table 1). For LiMgN, for example, a possibly disordered cation sublattice^109^ may lower the band gap compared to that of an ordered one assumed here. The disorder may introduce band tails, providing a plausible explanation for why the band gap calculated using HSE06 is markedly higher than that found experimentally. Indeed, for LiMgN all functionals are consistently markedly higher than expected from their MAE. For the other ternary compounds too, the presence of disorder may affect the band gap as well as the accumulation of small errors arising from, for example, exciton binding energies and temperature. Moreover,if exciton binding energies were incorporated in our calculations, the agreement could have been slightly better for phases where these have not been estimated experimentally since their inclusion generally decreases the band gap. However, the difference in the fundamental band gap vs the optical band gap is expected to be <0.1 eV although it is worth bearing in mind that some wide band gap nitrides such as Si_3_N_4_ can have an exciton energy of 0.3 eV or even higher^102^ as discussed above. Uncertainties in the estimated experimental band gap for ZnSnN_2_ may also explain the discrepancy between HSE06/mBJ and experiments. Large discrepancies in the gap between different experiments are probably due to the presence of extensive excess charge carriers (Burstein–Moss shift) and/or configurational disorder associated with kinetic trapping during deposition.^117^

For several wide band gap III-N phases, HSE06 systematically *underestimates* the experimental band gaps. For all AlN (zinc-blende), AlN (wurtzite), and BN (cubic/zinc-blende), HSE06 values are lower by 0.80, 0.67, and 0.48 eV, respectively, compared to experiment. The tendency of HSE06 to underestimate gaps for these wide band gap semiconductors and insulators is consistent with findings from previous benchmarks of inorganic compounds^5,15,30^ and is probably due to electronic overscreening caused by using a too low amount of exact Fock exchange (25% for HSE06). Increasing the fraction of Fock exchange to more than 30% for both AlN zinc-blende and wurtzite phases was necessary to reproduce the fundamental band gap measured experimentally.^134^

Although our HSE06 gap for AlN (wurtzite) is in good agreement with previous HSE06 calculations (where *E*_g_ = 5.64 eV^135^), the underestimation compared to experiment requires a comparison with higher levels of theory. Our calculated value of *E*_g_ using *G*_0_ *W*_0_@PBE is 5.99 eV (Table 3)—although this is in much better agreement with the fundamental band gap estimated experimentally (6.0-6.3 eV) compared to HSE06—it is higher than that calculated in a previous *G*_0_ *W*_0_@PBE study (where *E*_g_ is 5.72 eV).^29^ Moreover, if hybrid functionals (*G*_0_ *W*_0_@HSE) are used as starting points for quasiparticle band gap calculations, the gaps increase to 6.35 eV and 6.55 eV (*G*_0_ *W*_0_@PBE0). Fully self-consistent sc*GW* increases the band gap further to 6.8 eV,^158^ thus slightly worsening the agreement with experiment. This is expected from sc*GW* without the inclusion of vertex corrections in *W*. Also for AlN (zinc-blende), where the HSE06 gap appears to be too low, results from *GW* calculations are in overall better agreement with experiment, but the discrepancy between different *GW* results spans more than 0.5 eV depending on the flavor of *GW.* ^28,136^ More recent sc*GW* results^137^ overestimate the gap by only 0.3 eV, but again vertex corrections are not incorporated, which are required for a critical comparison with experiment.

Similarly for BN, although our calculated *G*_0_ *W*_0_@PBE (*E*_g_ = 6.27 eV for the cubic phase) is consistent with the *G*_0_ *W*_0_ results reported in ref 138 (which is 6.18 eV), the band gaps calculated using sc*GW* are larger for both this as well as the other BN polymorphs^138^ worsening the agreement with band gaps measured experimentally. Inclusion of vertex correction in *W* improves the band gap of cubic BN to 6.66 eV, but the value is still slightly higher than the experimental one.^30^ This illustrate that attempts to improve the calculated band gaps for III–V nitrides beyond those of the popular *G*_0_ *W*_0_@DFT schemes still remains a challenge to theory. On the other hand, the systematic cancellation of nonself-consistency and vertex corrections is reasonable well understood, pointing to *G*_0_ *W*_0_@DFT as a pragmatic choice to accurate band gaps, but a $∼O\left(N^{4}\right)$ increase in computational efforts with system size still hampers the method for systems with more than 20 atoms, suggesting that mBJ and HSE06 are the best candidates for the calculations of accurate band gaps.

Turning to the ternary AlGaN_2_, InGaN_2_, and AlInN_2_, the experimental gaps have been calculated from those of the end-members using an appropriate bowing parameter, whereas we used the PBE-optmized geometries reported in the Materials Project.^118^ Since GGA typically overestimates volumes and bond lengths by a few percentage, we carried out optmizations of these three compounds using HSE06 (which, in general, gives equilibrium volumes in much better agreement with experiment, with volumes differing by typically less than 1%). For InGaN_2_, for example, cell volumes decreased by 4% after reoptimization of the PBE optimiszed geometry using HSE06, whereas the band gaps calculated at the HSE06 geometries increased markedly by approximately 0.2 eV for all functionals.. The use of optmizied geometries from higher-level theory (e.g., HSE06) rather than LDA/PBE is important when crystallographic data from experiments are not available to allow for a critical comparison of band gaps from theory and experiment.

Although both mBJ and HSE06 tend to underestimate band gaps for some large/wide band gap semiconductors and possibly overestimate for some ternary Li-nitrides, the overall agreement with experiment is good, as reflected in the statistics collected in Table 3 and visualized in Figure 1. For materials with a band gap below ∼2 eV—possibly suitable for solar cell materials—most (80%) of the materials deviate by less than 0.3 eV from those measured experimentally. Possible sources of the discrepancy may be exciton binding energies (when not taken into account) and temperature effects (since we often compare with experiments conducted at room temperature).

### 3.3 Comparison of Benchmarks over Nitrides with Other Inorganic Material Classes

It is interesting to compare the performance of the functionals here with that of Borlido et al. since their data set—taken to a large extent from the compilation of Madelung^139^—contains very few pnictogenides but many chalcogenides and halides. The plot of MAE vs MAPE (Figure 2) confirms that LDA and PBE show very similar performance when compared to other inorganic materials, but all HLE16, mBJ, and HSE appear to perform better for nitrides. The lower MAPE of nitrides can, in part, be explained by the distribution of band gaps since the average band gap of the data set in ref 15 is around 2 eV, whereas the average band gap for the nitride data set in this work is higher (3 eV). Since band gaps are systematically too low in the entire gap range—illustrated by the linear fit of, e.g., the HLE16 data set (*a*_HLE16_ = −0.229, *b*_HLE16_ = 0.936, σ = 0.4), which is nearly parallel to *y* = *x* – materials with low gaps will have a higher weight in MAPE. The much lower absolute error, MAE for mBJ and HSE is surprising but may stem from bias and/or the quality of the data set. Our data set contains very few *f* and *d* elements, suggesting possibly a “*sp*-biased” data set compared to that of the large-scale benchmark of refs 5 and 15. However, Borlido et al.^15^ find very similar performance of the functionals when dividing the data set into subdata sets containing only *sp*, *d,* or *f* elements (with or without inclusion of spin-orbit coupling). It is, therefore, unlikely that the lower MAE for some of the functionals is due to any such bias. Here, we carefully and critically examine all nitride band gaps and exclude those expected to be hampered by systematic errors in the measurements.

**Figure 2:**
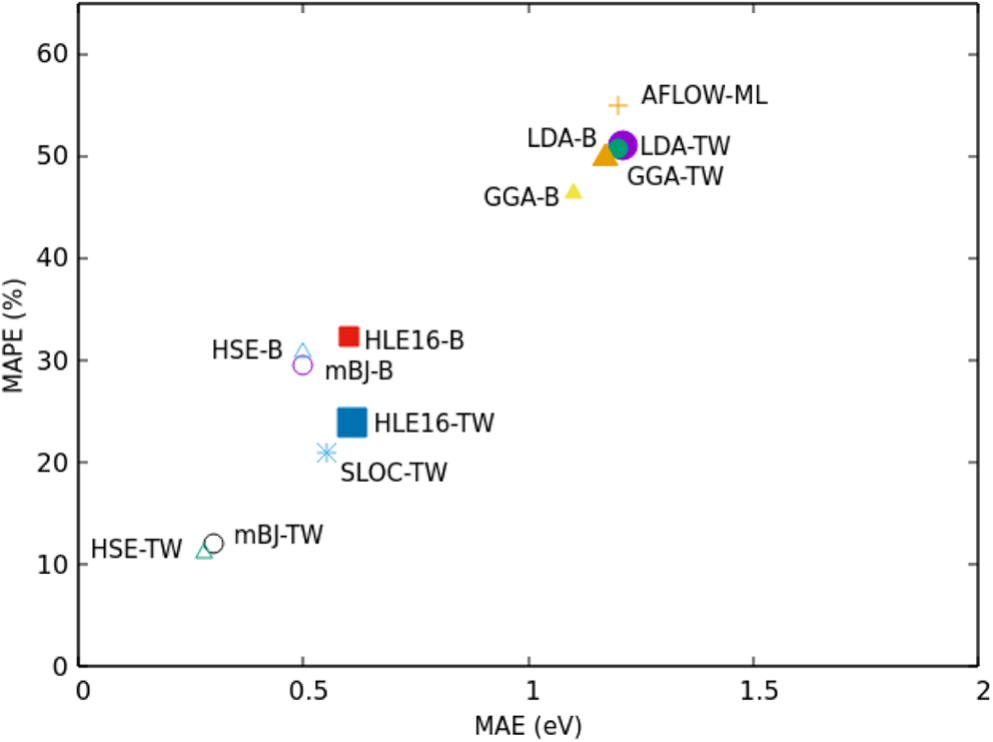
Comparison of the performance of the different functionals. The label “TW” refers to “This Work,” whereas “B” refers to a large-scale DFT benchmark reported by Borlido et al.^15^.

### 3.4 Choice of Functionals for High-Throughput Material Screening

The performance of the different functionals can also help develop strategies and selection metrics for high-throughput large-scale screening of materials for use in, e.g., solar cells. These strategies typically involve scanning (crystallographic) databases for candidate materials and calculating the band gap using an appropriate functional. Balance between cost and accuracy is, therefore, crucial when choosing a suitable DFT functional for large-scale materials screening. Since PBE and LDA severely underestimate band gaps, with many semiconductors being predicted to be semimetals and metals, these functionals are unsuitable for a preliminary screening of candidate materials. All SLOC, AK13LDA, and HLE16 also underestimate the gap, but in contrast to conventional LDA and GGA, none of the semiconductors are predicted to be semimetals or metals. Moreover, they are marginally more expensive than LDA/PBE and computationally much cheaper than meta-GGA-derived potentials such as TASK and mBJ (see Table 2).

HSE06 is the most expensive functional due to the costly screened Fock term. Although mBJ is more than an order of magnitude less expensive than HSE06, its overall similar accuracy explains its popularity. However, the two meta-GGA-derived functionals TASK and mBJ often suffer from slow convergence of the wave function, but this is not the case for HLE16 and AK13LDA. We can improve the slow convergence of the TASK and mBJ methods using PAW potentials that are better developed for the specific functional used. Although the *GW* potentials are developed to ensure accurate quasiparticle energies by reproducing atomic scattering properties over a wider energy range compared to conventional pseudopotentials, we find that the wave function of mBJ and TASK often converges faster using these potentials. However, TASK and mBJ are still one order of magnitude more expensive than HLE16 and AK13LDA. A possible strategy to develop a selection metric for large-scale material screening could be to scale HLE16/AK13LDA band gaps using $E_{g}^{s}=E_{g}+\Delta_{g}$ where Δ_g_ is the difference between a linear fit to experimental (benchmark) data and a linear fit to those calculated using a given functional (see Figure 1). For AK13LDA, for example, the MAPE decreases from 23% to about 15%, pointing to a future strategy for low-cost large-scale material screening, but 15% is still markedly higher than the MAPE from mBJ.

Recent development of property predictors using machine learning (ML) trained with input from large databases also provides a highly promising, low-cost strategy for estimating band gaps.^140^ We therefore predict band gaps for our data set using the AFLOW machine-learning algorithm^141^ where *ab initio* data from the AFLOW repository is used as input to develop materials structure–property predictors. However, the band gaps used as input for the ML training are typically calculated at the PBE levels, with only a small fraction of SCAN or hybrid-level data providing a poor-quality data set to train the predictor, severely hampering the accurate calculation of band gaps. Indeed, AFLOW-ML significantly underestimates the band gaps with an MAE of 1.40 eV (MAPE of 55%) and is also apparently unable to estimate band gaps for a few compounds with large unit cells. Developing and updating of databases with accurate band gaps is a prerequisite to predicting accurate band gaps, but databases with high-quality data are currently under development^142,143^ and typically limited to specialized sets.^144^

If large computational resources are available and accurate data are needed, a suitable strategy to predict new materials for solar cells would involve calculating band gaps using HSE06, possibly in conjunction with mBJ, as they may, in some cases, provide a complementary description of the electronic structure of nitrides. HSE06 tends to underestimate band gaps for wide band gap semiconductors because a 25% Fock exchange often exaggerates electronic screening.^15,132,145^ By contrast, mBJ is, by construction, less hampered by band gap underestimation^5,15,133^ and often more accurate for wide band gap materials. Indeed, the choice of functional for high-throughput screening depends on the performance in the target range, but our data set is too small to allow for a detailed analysis of the performance of the different functionals in particular subgap regions.

For systems with a small unit cell, *GW* calculations are, in general, more accurate than HSE, and single-shot *G*_0_ *W*_0_@PBE or *G*_0_ *W*_0_@HSE provide surprisingly accurate pragmatic approaches (in particular for *sp* systems) due to the well-understood systematic cancellation of errors explained by the lack of self-consistency and vertex corrections. For a critical examination of accurate experimental band gaps, fully consistent *GW* calculation levels incorporating vertex corrections, together with suitable pseudopotentials developed for GW calculations, are needed. However, these are only available for systems with a few atoms in the unit cell and challenging to implement for unsupervised large-scale screening even if computational resources were available. Moreover DFT is, by construction, unable to provide a systematic route to recover the “exact” exchange-correlation energy. By contrast, wave function-based theory allows for a systematic expansion toward the full configuration interaction wave function in a complete one-electron basis via, e.g., coupled cluster (CC) expansions in singles, doubles, triples, etc., or multiconfigurational expansions.^146^ Recent promising implementations of coupled cluster methods using Gaussian-based orbitals^147,148^ and the equation-of-motion CC method^148,149^ for the accurate calculation of band gaps point to promising future avenues, but CCSD and CCSD­(T) typically scale as $O\left(N^{5}\right)$ and $O\left(N^{7}\right)$ , respectively, and suffer from slow convergence with the size of the supercell and *k*-mesh which limits their application to the smallest systems. The development of theory that approaches chemical accuracy for larger systems also points to dielectric-dependent hybrid, DDH, functionals,^20,21,150^ where the dielectric constant is used to determine the fraction of exact exchange. The system-dependent DDH functionals are a natural generalization of conventional hybrid functionals toward GW and represent a promising $O\left(N^{3}\right)$ future avenue to accurate band gaps without resorting to quasiparticle $O\left(N^{4}\right)$ theory or beyond. In particular, the systematic overscreening of many wide band gap materials using HSE06 points to DDH as the next ladder. DDH can be further developed without empirical parametrization, and a benchmark of a recent implementation on metal oxides, including many notoriously difficult transition metal oxides, has an MAPE of only 7.4%.^150^

For large-scale strategies for material screening, mBJ remains an accurate and reasonable low-cost functional consistent with previous (large-scale) benchmarks^5,6^ but the slow convergence of the wave functions hampers its performance compared to e.g., AK13LDA/HLE16. The advantage of mBJ, however, compared to other promising “designed” potentials, such as HLE16 and AK13, is that mBJ has been extensively tested for a range of material classes, so its performance is well established. The well-tested semilocal GLLB family of exchange-correlation potentials, such as GGLB-SC^17,151^ , are comparable in accuracy to mBJ as demonstrated by, for example, the accurate low-cost calculations (MAE = 0.6 eV) of band gaps for a test set of 75 reference solids,^6^ which is similar to that of mBJ and HSE,^6^ providing possibly a reasonable alternative to mBJ for large-scale predictions.

Low-cost strategies for large materials screening point to AK13LDA/HLE16—which is only marginally slower than LDA and PBE—possibly in conjunction with scaling strategies to improve ME/MAE andMPE/MAPE—but previous extensive benchmark highlights that AK13 may be hampered by a lack of transferability and, therefore, less suitable for 3*d* transition metals.^5^

## 4 Conclusions

We have carried out a benchmark of exchange-correlation functionals for the calculation of fundamental band gaps of inorganic nitrides. Our motivation has been 4-fold. First, nitrides have not previously been benchmarked *per se* and are strongly under-represented in previous large-scale materials benchmarks. Incorporating nitrides in data sets for benchmarking theory is essential to ensure the transferability of functionals and to understand their strengths and weaknesses. Second, nitride semiconductors show promise as materials in a range of fields, including photovoltaics, energy storage, sensing, and detection. Although this has prompted an upsurge of interest in the prediction of new stable and metastable phases, nitrides remain surprisingly little explored both computationally and experimentally. Hence, an accurate prediction and compilation of their properties (including the band gap) can help guide future research. Third, further development of highly parametrized and designed potentials, such as AK13,^24^ benefits from large pools of band gaps to maximize accurate predictions. Fourth, the development of machine learning and strategies^154^ for band gap prediction requires large databases, in which the current database will contribute to a future large-scale balanced database required for benchmarking theory and for developing machine learning.

From a literature survey, we have therefore collected 25 binary nitrides and 11 ternary nitrides with a focus on nonmagnetic semiconductors. The data set incorporates elements spanning the periodic table, with all ionic, covalent, and metallic bonding environments being represented. Although our benchmark would benefit from a larger data set of band gaps, the lack of accurate data remains a drawback that, hopefully, can motivate future experimental work. Many measurements are carried out on thin, often nonstoichiometric, films where optical band gaps are either identified using Tauc plots or from an inspection of the shape without a critical evaluation of uncertainties and/or the underlying approximations made using such models. This hampers a direct comparison with *ab initio* theory, where fundamental band gaps are calculated on periodic systems representing bulk single crystals. Inspired by previous extensive benchmark tests, where 22 functionals have been tested in large-scale benchmarks,^5^ we select 8 of these, including well-established exchange-correlation functionals as well as promising new functionals developed to overcome the challenge with discontinuity in the exchange-correlation potentials which severely hampers conventional Kohn–Sham theory. That is, in addition to LDA and PBE, we investigated the performance of the simple Slater exchange functional, the parametrized LDA/GGA-derived high local exchange (HLE16), Armiento–Kümmel semilocal (AK13) functional, meta-GGA functionals including TASK and the modified Becke-Johnson functional (mBJ), as well as the Heyd–Scuseria–Ernzerhof (HSE06) hybrid functional. For selected compounds, we also carry out quasiparticle *GW* calculations to better assess experimental band gaps.

Consistent with previous extensive benchmark tests, conventional LDA and PBE unsystematically and largely underestimate band gaps with MAE (MAPE) of 1.22 (51%) and 1.17 (50%) respectively. This is also illustrated in Figure 2 where we compare MAE/MAPE with those reported in a previous large-scale benchmark.^15^ The SLOC, HLE16, and AK13LDA functionals all show improvement over LDA/PBE with MAE of 0.55, 0.62, and 0.51 eV (MAPE 21.0%, 23.2%, and 22.9%). Moreover, all five compounds predicted to be semimetals or metals by PBE and LDA were found to be semiconductors. mBJ and HSE06 are the most accurate with MAE = 0.30 and 0.28 eV (MAPE 12.1% and 11.1%), respectively. For 80% of the compounds, the difference between the gaps calculated using HSE06/mBJ and experiment was less than 0.5 eV and for compounds where the deviation is larger than 0.5 eV, we have investigated possible explanations for the deviation between theory and experiment.

Our results point to different strategies for large-scale material screening depending on the accuracy required and the available computational resources. Low-cost strategies suggests using AK13LDA (or HLE16) possibly with a scaling-of-the-gaps equation to reduce MAPE. The possibility of further developing the AK13 family of functionals could be a promising future route. mBJ remains a superior approach for a medium-cost strategy, giving highly accurate band gaps. Although HSE06 is substantially more expensive than mBJ, it provides a robust, general, well-tested, and accurate approach, particularly when other relevant properties are needed, such as dielectric constants and absorption spectra. Moreover, different implementations of HSE06 can markedly affect the overall CPU time; for example, if Gaussian-type orbitals may speed up the calculation of exact exchange. The next level of accuracy beyond mBJ and HSE06 points to the pragmatic single-shot *G*_0_ *W*_0_ method, which generally predicts gaps, at least for *sp* compounds, to chemical accuracy but is limited to about 20 atoms. Fully sc*GW*, including vertex correction, is required for a critical examination of accurate experiments without being hampered by the systematic cancellation of errors. Recent development in property predictors using machine learning (ML), such as the AFLOW-ML algorithms trained with input from large databases, is promising low-cost strategy for estimating band gaps. However, the band gaps used as input for the ML training are typically calculated at the PBE level, providing a poor-quality data set to train the predictor, and hence the predicted gaps are strongly underestimated. The development of large databases including nitrides with accurate band gaps is therefore a prerequisite for predicting accurate band gaps.
